# Gating of NMDA receptor-mediated hippocampal spike timing-dependent potentiation by mGluR5

**DOI:** 10.1016/j.neuropharm.2012.05.021

**Published:** 2012-09

**Authors:** Jeehyun Kwag, Ole Paulsen

**Affiliations:** a Department of Brain and Cognitive Engineering, Korea University, 145 Anam-Ro, Seongbuk-Gu, Seoul 136-701, Republic of Korea; b Department of Physiology, Development and Neuroscience, University of Cambridge, Cambridge, UK

**Keywords:** Hippocampus, mGluR5, Spike timing-dependent plasticity, CA1, Development, Rat

## Abstract

Hippocampal long-term potentiation (LTP) is believed to be important for learning and memory. Experimentally, the pairing of precisely timed pre- and postsynaptic spikes within a time window of ∼10 ms can induce timing-dependent LTP (tLTP), but the requirements for induction of tLTP change with development: in young rodents single postsynaptic spikes are sufficient to induce tLTP, whereas postsynaptic burst firing appears to be required in the adult. However, hippocampal neurons *in vivo* show theta-modulated single spike activities also in older hippocampus. Here we investigated the conditions for single spike pairing to induce tLTP at older CA3–CA1 synapses. We found that the pairing of single pre- and postsynaptic spikes could induce tLTP in older hippocampus when the postsynaptic neuronal membrane was depolarized and the pairing frequency exceeded ∼4 Hz. The spike frequency requirement is postsynaptic, as tLTP could still be induced with presynaptic stimulation at 1 Hz as long as the postsynaptic spike frequency exceeded ∼4 Hz, suggesting that postsynaptic theta-frequency activity is required for the successful induction of tLTP at older CA3–CA1 synapses. The induction of tLTP was blocked by an NMDA receptor antagonist and by the selective mGluR5 blockers, MPEP and MTEP, whereas activation of mGluR1 and mGluR5 by DHPG relieved the postsynaptic spike frequency requirement for tLTP induction. These results suggest that activation of mGluR5 during single-spike pairing at older CA3–CA1 synapses gates NMDA receptor-dependent tLTP.

## Highlights

► Single-spike pairing can induce tLTP at older hippocampal CA3–CA1 synapses. ► tLTP induction requires postsynaptic depolarization and postsynaptic spike rate >4 Hz. ► tLTP induction requires NMDA receptors and is gated by mGluR5.

## 1 Introduction

Synaptic plasticity is believed to be important for hippocampus-dependent learning and memory processes (Bliss and Collingridge, 1993; Bliss and Lomo, 1973; Martin et al., 2000; Morris et al., 1986). A computationally attractive synaptic learning rule that could contribute to behavioral learning and memory is spike timing-dependent plasticity (STDP) (Caporale and Dan, 2008; Markram et al., 2011), in which the direction of plasticity is governed by the order of firing in pre- and postsynaptic neurons. Timing-dependent long-term potentiation (tLTP) is induced when the presynaptic neuron fires before the postsynaptic neuron within a time window of 10–20 ms whereas the reverse spike order can induce timing-dependent long-term depression (tLTD). STDP has been demonstrated in primary cultures of dissociated hippocampal neurons (Bi and Poo, 1998), hippocampal organotypic culture (Debanne et al., 1998), and in juvenile hippocampal slices (Kwag and Paulsen, 2009; Meredith et al., 2003). Interestingly, in hippocampal slices prepared from rodents more than 3 weeks old, tLTP induction with single-spike pairing failed and instead, postsynaptic bursts appeared to be required (Buchanan and Mellor, 2007; Meredith et al., 2003; Pike et al., 1999; Wittenberg and Wang, 2006). This developmental change has been attributed to maturation of GABA_A_ receptor-mediated inhibition (Meredith et al., 2003). Thus, back-propagating action potentials (bAP) (Stuart and Sakmann, 1994), which act as a coincidence signal during NMDA receptor-mediated tLTP induction, propagate reliably in dendrites of juvenile CA1 pyramidal neurons (Spruston et al., 1995), but are attenuated by dendritic GABA_A_ receptor-mediated inhibition (Tsubokawa and Ross, 1996), which increases with development in the first few postnatal weeks (Banks et al., 2002). Moreover, block of GABA_A_ receptors restores the ability of single postsynaptic spikes to induce tLTP in adult rat (Meredith et al., 2003). In addition to GABAergic maturation, developmental changes in signaling cascades (Yasuda et al., 2003), transmitter release (Bolshakov and Siegelbaum, 1995) and metabotropic glutamate receptor (mGluR) function (Huber et al., 2000) are also known to influence the induction of synaptic plasticity, suggesting additional factors that might contribute to the developmental switch in tLTP induction.

Although spike burst activity is seen in some hippocampal CA1 pyramidal neurons during theta activity *in vivo*, individual CA1 pyramidal neurons also exhibit single spikes in ensemble recordings from adult hippocampus (Frank et al., 2001; Kuang et al., 2010). Thus, a requirement of postsynaptic bursts would limit the occurrence of tLTP in older hippocampus. To address this issue, we therefore investigated under what conditions tLTP could be induced with single-spike pairing in older hippocampal slices. We found that the combination of postsynaptic depolarization and theta-frequency spiking activity in the postsynaptic neuron enabled a single-spike pairing protocol to induce tLTP. In addition, we found that activation of a metabotropic glutamate receptor, mGluR5, is necessary for single-spike pairing to induce tLTP. We propose that mGluR5 gates tLTP in older hippocampus, suggesting a functional role of mGluR5 in NMDA receptor-dependent tLTP.

## 2 Methods

### 2.1 Slice preparation and electrophysiology

All procedures were carried out in accordance with the UK Animals (Scientific Procedures) Act (1986) under personal and project licenses issued by the Home Office. Horizontal hippocampal slices (350 μm) were prepared from 21-35-day-old Wistar rats after decapitation under deep isoflurane-induced anesthesia. The brain was removed and slices were cut with a microtome (VT1000S or VT1200S, Leica) in an ice-cold oxygenated artificial cerebrospinal fluid (ACSF) containing (mM): NaCl 126; KCl 3; NaH_2_PO_4_ 1.25; MgSO_4_ 2; CaCl_2_ 2; NaHCO_3_ 25; glucose 10; pH 7.2–7.4; bubbled with carbogen gas (95% O_2_, 5% CO_2_). Slices were maintained at room temperature (22–25 °C) in a submerged-style holding chamber with ACSF for at least 1 h before being transferred to the recording chamber, which was perfused with oxygenated ACSF maintained at a temperature of 28–33 °C. Hippocampal CA1 pyramidal neurons were identified by infrared differential interference contrast video microscopy (Sakmann and Stuart, 1995) (Axioskop FS, Zeiss). Whole-cell patch-clamp recordings were performed using Multiclamp-700B (Molecular Devices) in either current-clamp mode or in voltage-clamp mode with patch pipettes pulled from standard-walled borosilicate glass (tip resistance 6–12 MΩ for current-clamp, 3–5 MΩ for voltage-clamp). In current-clamp experiments, the pipette solution contained (mM): Potassium gluconate 110; HEPES 40; NaCl 4; ATP-Mg 4; GTP 0.3 (pH 7.2–7.3; osmolarity 270–300 mosmol/l) and in voltage-clamp experiments, the pipette solution contained (mM): CsCl 140; HEPES 10; ATP-Mg 2; GTP 0.3; QX-314 5 (pH 7.2, osmolarity 280–290 mosmol/l).

All recordings were low-pass filtered at 2 kHz and acquired at 5–8 kHz using ITC-18 AD board (HEKA) and Igor Pro software (WaveMetrics). Igor Pro software was used for generating command signals, acquiring data as well as data analysis. In current-clamp recordings, only cells with resting membrane potential negative to −55 mV and with input resistance in the range of 100–200 MΩ were included in the analysis. In voltage-clamp recordings, 10 min were allowed after break-through for stabilization before recordings commenced. Series and input resistance was monitored throughout the experiment and cells with >20% change in series resistance were discarded.

### 2.2 tLTP induction protocol

**Fig. 1:**
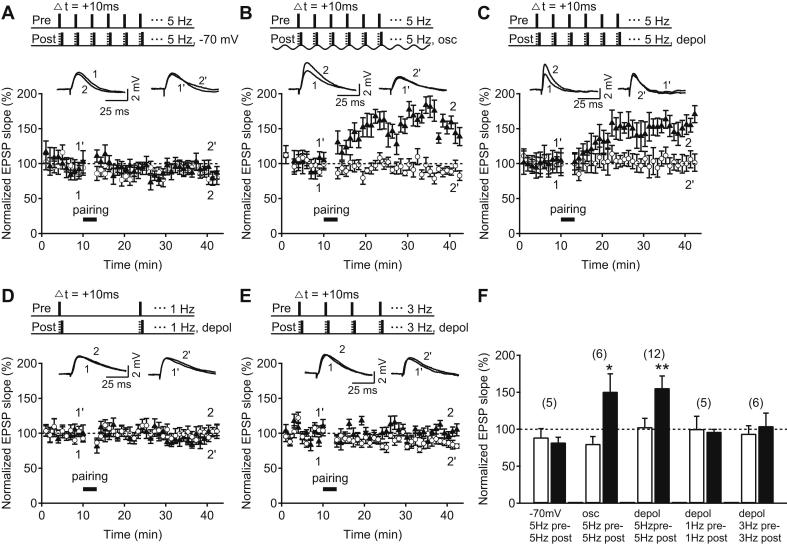
tLTP induction by pairing single pre- and postsynaptic spikes at older hippocampal synapses requires postsynaptic membrane depolarization and pairing at theta frequency. (A–C) Top, Pairing protocols in which 5 Hz Schaffer collateral stimulation (pre) was paired with 5 Hz CA1 pyramidal neuron spikes (post) elicited by 3 ms depolarizing current steps from −70 mV (A), by theta-like postsynaptic membrane potential oscillation (osc, B) and by 3 ms depolarizing current steps from membrane potential near threshold (depol, C). Δ*t* denotes time interval between pre- and postsynaptic neuronal activation. Sample EPSP traces before (test: 1, control: 1′) and after the pairing (test: 2, control: 2′). Time course of the normalized Schaffer collateral EPSP slope of control (open circle) and test pathway (black triangles) before and after each pairing protocol. All pairings were repeated 200 times. (D–E) Top, Pairing protocol as in (C) but with pairing repeated at 1 (D) and 3 Hz (E). Time course of the normalized Schaffer collateral EPSP slope of control (open circle) and test pathway (black triangles) before and after each pairing protocol. (F) Summary of results 25–30 min after each pairing protocol in control (white bar) and test pathway (black bar). Error bars are SEM; *p < 0.05; **p < 0.01, Student's *t* test. The number of experiments is shown in parentheses.

**Fig. 2:**
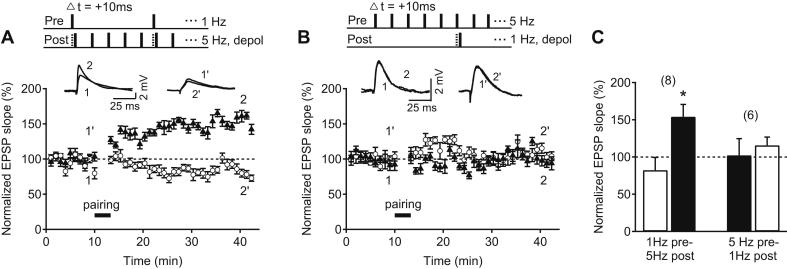
Postsynaptic spiking at theta-frequency is sufficient for single-spike pairing-induced tLTP at older hippocampal synapses. (A) Top, Pairing protocols in which 1 Hz Schaffer collateral stimulation (pre) was paired with 5 Hz CA1 pyramidal neuron spikes (post) elicited by 3 ms depolarizing current steps from membrane potential near threshold (depol, A). Δ*t* denotes time interval between pre- and postsynaptic neuronal activation. Sample EPSP traces before (test: 1, control: 1′) and after the pairing (test: 2, control: 2′). Time course of the normalized Schaffer collateral EPSP slope of control (open circle) and test pathway (black triangles) before and after each pairing protocol. (B) Top, Pairing protocols in which 5 Hz Schaffer collateral stimulation (pre) was paired with 1 Hz CA1 pyramidal neuron spikes (post) elicited by 3 ms depolarizing current steps from membrane potential near threshold (depol, B). Δ*t* denotes time interval between pre- and postsynaptic neuronal activation. Sample EPSP traces before (test: 1, control: 1′) and after the pairing (test: 2, control: 2′). Time course of the normalized Schaffer collateral EPSP slope of control (open circle) and test pathway (black triangles) before and after each pairing protocol. (C) Summary of results 25–30 min after each induction protocol in control (white bar) and test pathway (black bar). Error bars are SEM; *p < 0.05, Student's *t* test. The number of experiments is shown in parentheses.

**Fig. 3:**
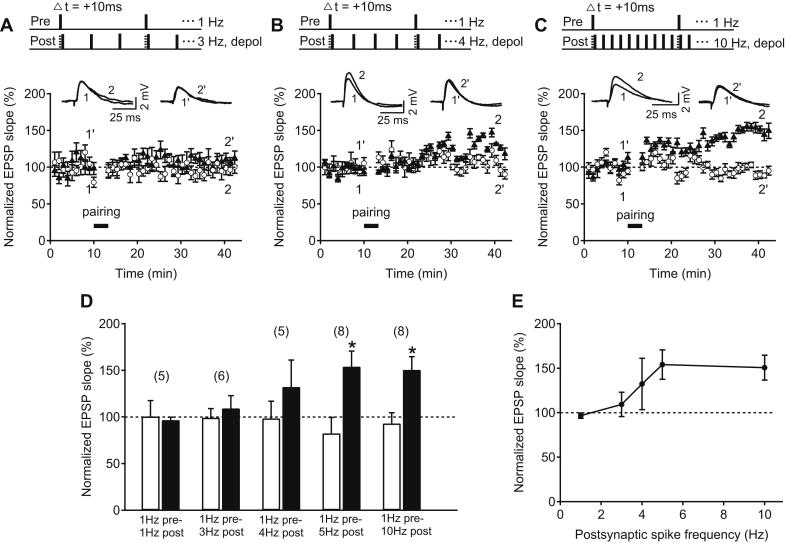
Single-spike pairing-induced tLTP at older hippocampal synapses depends on postsynaptic spike frequency. (A–E) Top, Pairing protocols in which Schaffer collateral stimulation at 1 Hz (pre) was paired with CA1 pyramidal neuron spikes (post) at 3 (A), 4 (B) and 10 Hz (C) by 3 ms depolarizing current steps from membrane potential near threshold (depol). Δ*t* denotes time interval between pre- and postsynaptic neuronal activation. Sample EPSP traces before (test: 1, control: 1′) and after the pairing (test: 2, control: 2′). Time course of the normalized Schaffer collateral EPSP slope of control (open circle) and test pathway (black triangles) before and after each pairing protocol. (D) Summary of results 25–30 min after each pairing protocol in control (white bar) and test pathway (black bar). Error bars are SEM; *p < 0.05, Student's *t* test. The number of experiments is shown in parentheses. (E) Mean normalized EPSP slope 25–30 min after each pairing protocol plotted against the postsynaptic spike frequency. Error bars are SEM.

**Fig. 4:**
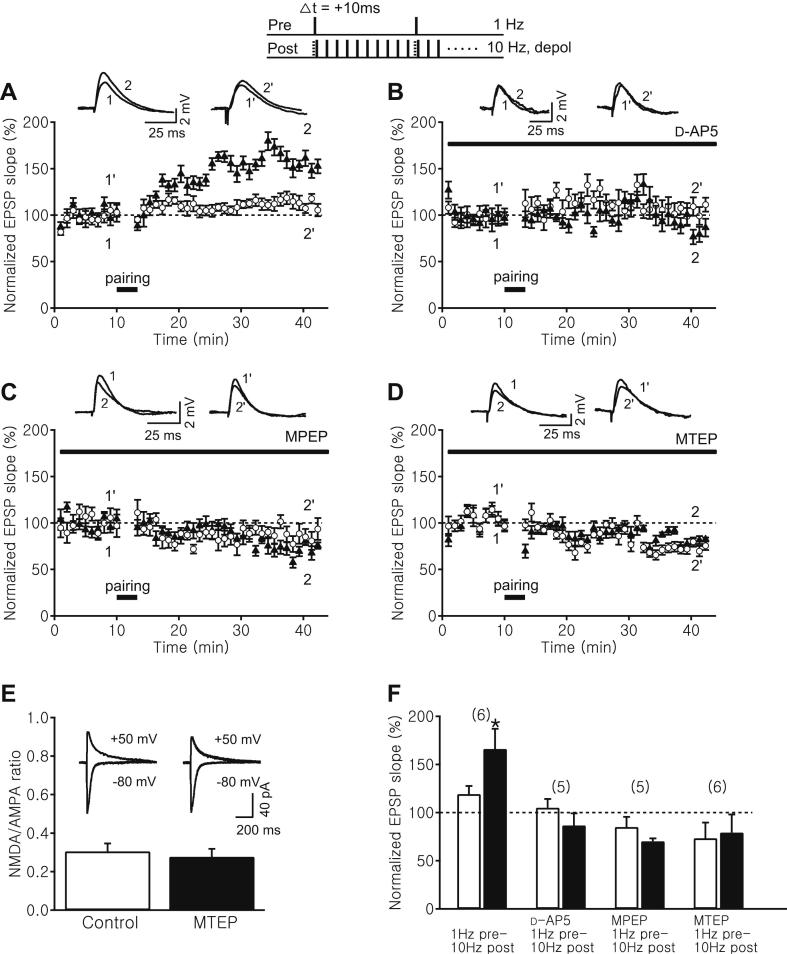
mGluR5 antagonists block the induction of tLTP at older synapses. Top, Pairing protocols in which Schaffer collateral stimulation at 1 Hz (pre) was paired with CA1 pyramidal neuron spikes (post) elicited at 10 Hz by 3 ms depolarizing current steps from membrane potential near threshold (depol). Δ*t* denotes time interval between pre- and postsynaptic neuronal activation. Sample EPSP traces before (test: 1, control: 1′) and after the pairing (test: 2, control: 2′). (A–D) Time course of the normalized Schaffer collateral EPSP slope of control (open circle) and test pathway (black triangles) before and after each pairing protocol in control condition (A), and in the presence of 50 μM d-AP5 (B), 10 μM MPEP (C) or 500 nM MTEP (D). (E) The NMDA/AMPA ratio in control condition and in the presence of 500 nM MTEP. Inset: sample current traces recorded in voltage-clamp mode at −80 mV and +50 mV. (F) Summary of results 25–30 min after each pairing protocol in control (white bar) and test pathway (black bar). Error bars are SEM; *p < 0.05, Student's *t* test. The number of experiments is shown in parentheses.

**Fig. 5:**
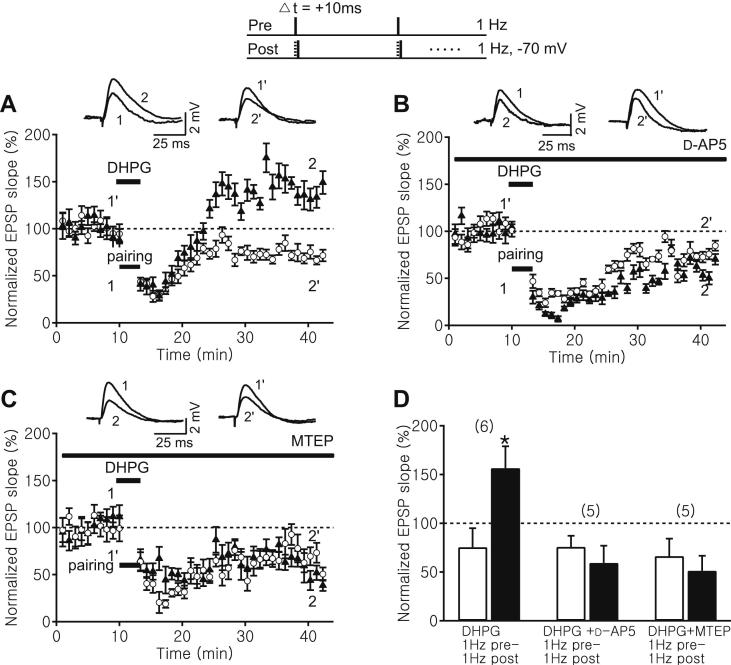
An mGluR agonist facilitates the induction of tLTP at older synapses. Top, Pairing protocols in which 1 Hz Schaffer collateral stimulation (pre) was paired with 1 Hz CA1 pyramidal neuron spikes (post) elicited by 3 ms depolarizing current steps from -70 mV. Δ*t* denotes time interval between pre- and postsynaptic neuronal activation. Sample EPSP traces before (test: 1, control: 1′) and after the pairing (test: 2, control: 2′). (A–C) Time course of the normalized Schaffer collateral EPSP slope of control (open circle) and test pathway (black triangles) before and after each pairing protocol with 50 μM DHPG applied during pairing (A), and in the presence of 50 μM d-AP5 (B) or 500 nM MTEP (C). (D) Summary of results 25–30 min after each pairing protocol in control (white bar) and test pathway (black bar). Error bars are SEM; *p < 0.05, Student's *t* test. The number of experiments is shown in parentheses.

Two stainless steel monopolar stimulating electrodes were positioned in the stratum radiatum either side of the recorded neuron. One electrode was used to stimulate the test pathway, the other to stimulate a control pathway. The test electrode was always positioned on the CA3 side of the neuron and closer to the soma than the control electrode (Buchanan and Mellor, 2007; Buchanan et al., 2010). Excitatory postsynaptic potentials (EPSPs) were evoked at 0.2 Hz with brief current pulses (20–40 μs, 80–300 μA) during baseline recording of 10 min. After a stable EPSP baseline was established with membrane potential held at about −70 mV by current injection, tLTP was induced by a pre-before-post pairing protocol whereby the start of the evoked EPSP was followed by a postsynaptic spike within 10 ms. Postsynaptic spikes were elicited either by a brief depolarizing current step (3 ms, 1000 pA) or by injecting a constant depolarizing current superposed on an oscillatory inhibitory conductance using dynamic clamp so that a spike occurred at the peak of each oscillatory cycle (see Kwag and Paulsen, 2009). All pairings were done within a time window (Δ*t*) of 10 ms between presynaptic stimulation and postsynaptic spike, except for the experiment shown in Fig. 1B in which the presynaptic input was stimulated 10 ms before the peak of the oscillation. To test the effect of pairing frequency on tTLP induction, pre-before-post pairing was carried out at 5 Hz (Fig. 1A–C), 3 Hz (Fig. 1E) and 1 Hz (Figs. 1D and 5A–C). In Fig. 2, EPSPs were evoked at 1 Hz and postsynaptic spikes were elicited at 5 Hz (Fig. 2A) or EPSPs were stimulated at 5 Hz with postsynaptic spikes elicited at 1 Hz (Fig. 2B). To test the effect of postsynaptic spike frequency on tLTP induction, EPSPs were stimulated at 1 Hz and postsynaptic spikes were elicited at 3–10 Hz (Fig. 3A–C, Fig. 4A–D). The membrane potential was depolarized to near threshold in all experiments except in Fig. 1A and Fig. 5 where it was kept at −70 mV. All pairings were repeated 200 times.

The EPSP was monitored for at least 30 min after the end of the pairing protocol and presynaptic stimulation frequency remained at 0.2 Hz. The slope of the EPSP was used as an index of synaptic efficacy, measured using a linear fit on the rising slope of the EPSP between time points corresponding to 20–25% and 75–80% of the EPSP peak amplitude during baseline condition. Changes in synaptic efficacy were estimated as percentage change relative to the mean EPSP slope during the 10-min baseline period. To compare synaptic efficacy between cells and between pairing paradigms, the mean of the normalized EPSP slope in the time period between 25 and 30 min after the end of the induction protocol was calculated.

### 2.3 NMDA/AMPA receptor-mediated current ratio

Voltage-clamp recordings of CA1 pyramidal neurons were performed in slices with afferents from the CA3 cut and 3 μM gabazine was added to block GABA_A_ receptor-mediated inhibition throughout the experiment. Excitatory synaptic currents in response to Schaffer collateral stimulation were recorded at −80 mV and +50 mV, repeated five times. AMPA receptor-mediated current (AMPA current) was measured as the peak current at −80 mV and NMDA receptor-mediated current (NMDA current) was estimated at +50 mV as the current amplitude 50 ms after the peak of AMPA current. The ratio between NMDA and AMPA current (NMDA/AMPA ratio) was calculated by dividing the estimated NMDA current by the peak AMPA current.

### 2.4 Drugs

All drugs were obtained from Tocris. d(−)-2-Amino-5-phosponopentanoic acid (d-AP5; 50 μM) was used to block NMDA receptors, SR95531 hydrobromide (gabazine, 3 μM) was added to block GABA_A_ receptors and (RS)-3,5-dihydroxyphenylglycine (DHPG; 50 μM) was used to activate group I mGluR. Either 2-methyl-6-(phenylethynyl)-pyridine (MPEP, 10 μM) or 3-((2-Methyl-1,3-thiazol-4-yl)ethynyl)pyridine hydrochloride (MTEP, 500 nM) was used to block mGlu5 receptors.

### 2.5 Statistical analysis

Significance of differences was tested with two-sample paired Student's *t* test and differences between experimental conditions were considered statistically significant when *p* < 0.05. Data are expressed as mean ± SEM.

## 3 Results

Induction of tLTP by pairing pre- and postsynaptic activity from resting membrane potential requires postsynaptic burst firing at older hippocampal Schaffer collateral-CA1 synapses (Buchanan and Mellor, 2007; Meredith et al., 2003; Pike et al., 1999; Wittenberg and Wang, 2006). Consistent with this, we found that pairing single presynaptic stimuli with single postsynaptic spikes at 5 Hz from resting or hyperpolarized membrane potential (−70 mV) failed to induce tLTP in 21–35-day-old rats (test pathway: 82.3 ± 6.9%, control pathway: 89.0 ± 11.7%, *n* = 5, *p* > 0.05, Fig. 1A, F). However, during theta rhythm *in vivo*, spiking postsynaptic CA1 pyramidal neurons are depolarized (Harvey et al., 2009). We therefore asked whether depolarization of the postsynaptic cell during induction would influence the tLTP induction requirements. Indeed, when presynaptic stimuli were paired with single postsynaptic spikes during theta-like oscillation, which produced an average postsynaptic membrane depolarization of 14.6 ± 0.7 mV, pre-before-post pairing at 5 Hz was sufficient to induce tLTP (test pathway: 150.9 ± 24.1%, control pathway: 80.3 ± 9.8%, *n* = 6, *p* < 0.05, Fig. 1B, F). Similarly, when the postsynaptic membrane was depolarized by constant current just below spike threshold without oscillation during the induction (depolarization of 12.7 mV ± 2.8 mV), pairing of single presynaptic stimuli 10 ms before single postsynaptic spikes at 5 Hz was sufficient to induce tLTP (test pathway: 155.7 ± 16.1%, control pathway: 103.0 ± 11.7%, *n* = 12, *p* < 0.01, Fig. 1C, F). Thus, postsynaptic neuronal membrane depolarization appears to be important for the induction of tLTP at older hippocampal CA3–CA1 synapses. In order to test whether the effect of postsynaptic neuronal membrane depolarization alone is sufficient to enable tLTP induction with single-spike pairing, we repeated pre-before-post single-spike pairing during postsynaptic depolarization at lower frequencies. However, under these conditions, tLTP was not induced. Both 1 Hz pairing and 3 Hz pairing failed to induce tLTP (1 Hz, test pathway: 96.6 ± 3.0%, control pathway: 100.4 ± 17.0%, *n* = 5, *p* > 0.05; 3 Hz, test pathway: 104.4 ± 17.4%, control pathway: 94.0 ± 10.7%, *n* = 6; *p* > 0.05; Fig. 1D–F). These results demonstrate that both depolarization of the postsynaptic neuronal membrane and a minimum repetition frequency of single-spike pairing, close to 5 Hz, are required for the induction of tLTP at older hippocampal synapses.

To investigate whether theta-frequency activity is required in both the presynaptic and the postsynaptic neuron during induction, or whether the frequency requirement relates to either the presynaptic or the postsynaptic side, we repeated the experiments using 1 Hz presynaptic stimulation and 5 Hz postsynaptic spiking or *vice versa* whilst keeping the total number of pairings the same (Fig. 2). When presynaptic stimulation was delivered at 1 Hz and the postsynaptic neuron fired at 5 Hz (1 Hz pre–5 Hz post) with postsynaptic membrane depolarized near threshold, tLTP was induced (test pathway: 154.0 ± 16.6%, control pathway: 82.4 ± 17.1%, *n* = 8, *p* < 0.05, Fig. 2A, C). In contrast, when the induction paradigm was reversed, i.e. presynaptic stimulation at 5 Hz paired with single postsynaptic spikes at 1 Hz with postsynaptic membrane depolarized near threshold (5 Hz pre – 1 Hz post), no tLTP was induced (test pathway: 102.3 ± 22.3%, control pathway: 115.7 ± 11.0%, *n* = 6, *p* > 0.05, Fig. 2B, C). Therefore, tLTP can be induced at older hippocampal CA3–CA1 synapses with single-spike pairing but it requires maintained postsynaptic depolarization as well as postsynaptic spiking near theta-frequency.

We next investigated the postsynaptic spike frequency-requirement for the induction of tLTP at older hippocampal synapses. Keeping the presynaptic stimulation rate at 1 Hz, the postsynaptic spike frequency was varied between 3 and 10 Hz (Fig. 3A–C). The postsynaptic neuronal membrane was depolarized to near threshold during all protocols. Postsynaptic spike firing at 1 Hz (as before; Fig. 1D), as well as 3 Hz and 4 Hz failed to induce statistically significant tLTP (3 Hz, test pathway: 109.3 ± 13.7%, control pathway: 99.2 ± 9.7%, *n* = 6, *p* > 0.05; 4 Hz, test pathway: 137.1 ± 28.8%, control pathway: 98.5 ± 18.4%, *n* = 5, *p* > 0.05, Fig. 3A–B, D). However, with postsynaptic spike rate at 5 Hz (1 Hz pre – 5 Hz post; Fig. 2A) and 10 Hz (1 Hz pre – 10 Hz post), robust tLTP was induced (10 Hz, test pathway: 150.5 ± 14.0%, control pathway: 93.0 ± 11.5%, *n* = 7, *p* < 0.05, Fig. 3C–D). In order to visualize the postsynaptic spike frequency-dependence of the induction of tLTP, the mean of the last 5 min of the normalized EPSP slope after pairing was plotted against the postsynaptic firing frequency during induction (Fig. 3E). This plot shows that tLTP emerges when the postsynaptic spike rate exceeds 3–4 Hz.

To confirm that the tLTP we observed with the induction paradigms in Fig. 3 is NMDA receptor-dependent, we repeated the 1 Hz pre – 10 Hz post induction paradigm in the control condition (Fig. 4A) and in the presence of the NMDA receptor antagonist, d-AP5 (50 μM), which completely blocked the induction of tLTP (test pathway: 87.6 ± 12.5%, control pathway: 105.1 ± 9.0%, *n* = 5, *p* > 0.05, Fig. 4B, F).

In addition to NMDA receptors, some forms of hippocampal LTP require the activation of a subtype of group 1 mGluR, mGluR5 (Bashir et al., 1993; Bortolotto et al., 1994; Chinestra et al., 1993; Francesconi et al., 2004). In order to test whether mGluR5 plays a role in the induction of tLTP, we repeated the experiment in the presence of the specific mGluR5 antagonist, MPEP (10 μM), which completely blocked the induction of tLTP and instead uncovered significant depression (test pathway: 70.2 ± 3.0%, control pathway: 85.0 ± 10.6%, *n* = 5, *p* > 0.05, Fig. 4C, F). Since MPEP has been reported to interfere with NMDA receptor function (Lea and Faden, 2006), we repeated the experiment using a more selective mGluR5 antagonist, MTEP. We found that 500 nM MTEP also completely blocked the induction of tLTP (test pathway: 79.3 ± 18.7%, control pathway: 73.4 ± 16.2%, *n* = 6, *p* > 0.05, Fig. 4D, F). To exclude the possibility that MTEP acts by altering NMDA receptor function, we performed voltage-clamp experiments and estimated the NMDA/AMPA ratios in control condition and in the presence of 500 nM MTEP. They were not significantly different (control: 0.31 ± 0.04, test: 0.28 ± 0.04, *n* = 5, *p* > 0.05, Fig. 4E), suggesting that MTEP at this concentration has no significant effect on synaptically activated NMDA current. These results show that the induction of tLTP at older hippocampal synapses requires NMDA receptors and suggest that tLTP induction is gated by the activation of mGluR5.

Activation of group I mGluR is known to depolarize the membrane and increase the excitability of CA1 pyramidal neurons (Clement et al., 2009; Gereau and Conn, 1995; Ireland and Abraham, 2002; Mannaioni et al., 2001). In order to test whether enhanced mGluR activation during induction could replace the postsynaptic depolarization requirement for tLTP, we applied the group 1 mGluR agonist, DHPG (50 μM), during the induction with single-spike pairing at 1 Hz. Application of DHPG soon caused a postsynaptic membrane depolarization of 9.8 ± 0.7 mV, which is comparable to the amount of depolarization necessary to induce tLTP (Fig. 1B–C). Holding the postsynaptic somatic membrane potential at −70 mV in the presence of DHPG, this pairing protocol induced tLTP (test pathway: 156.6 ± 22.3%, control pathway: 75.4 ± 19.4%, *n* = 6, *p* < 0.05, Fig. 5A, D), and the amount of potentiation was not significantly different from that induced by 1 Hz pre – 10 Hz post pairing without DHPG (Figs. 3C and 4A). Interestingly, the unpaired control pathway depressed following DHPG application, which is consistent with several previous reports using field recordings that mGluR activation can induce long-term depression (Fitzjohn et al., 1999; Palmer et al., 1997). Thus, in the presence of a group 1 mGluR agonist, single postsynaptic spikes following presynaptic stimulation were sufficient to convert synaptic depression into potentiation (Fig. 5A, D). Such tLTP induced in the presence of DHPG was NMDA receptor-dependent since no potentiation was observed in the test pathway in the presence of d-AP5 (50 μM), but both test and control pathways showed long-term depression (test pathway: 59.4 ± 17.6%, control pathway: 76.8 ± 11.4%, *n* = 5, *p* > 0.05, Fig. 5B, D). Lastly, to investigate whether such DHPG-mediated conversion of tLTD to tLTP is also gated by mGluR5, we repeated the experiment in the presence of the more selective mGluR5 antagonist, MTEP (500 nM). In the presence of MTEP, no potentiation was observed in the test pathway following pairing but both test and control pathways showed long-term depression (test pathway: 51.3 ± 15.2%, control pathway: 66.3 ± 17.9%, *n* = 5, *p* > 0.05, Fig. 5C–D).

## 4 Discussion

Here we investigated whether pairing single pre- and postsynaptic spikes at older hippocampal CA3–CA1 synapses could induce NMDA receptor-dependent tLTP. We found that a combination of postsynaptic membrane depolarization and postsynaptic spiking activity at a rate exceeding ∼4 Hz enabled tLTP induction using single pre- and postsynaptic spikes. In addition, we found that tLTP induction using single-spike pairing is gated by mGluR5 since blockade of mGluR5 prevented tLTP while activation of group 1 mGluR relieved the postsynaptic spike frequency requirement for tLTP induction.

Pairing of single pre- and postsynaptic spikes reliably induces tLTP at CA3–CA1 synapses in young rodents (Kwag and Paulsen, 2009; Meredith et al., 2003), but fails to induce tLTP in adults when postsynaptic burst activity seems to be required (Buchanan and Mellor, 2007; Meredith et al., 2003; Wittenberg and Wang, 2006). Consistent with these previous reports, we found that the single-spike pairing paradigm failed to induce tLTP at older CA3–CA1 synapses when the postsynaptic somatic membrane potential was held near −70 mV (Fig. 1A). However, we found that tLTP could be induced using single-spike pairing if three conditions were met: Postsynaptic depolarization, postsynaptic spikes at theta frequency, and activation of mGluR5.

Postsynaptic depolarization was necessary for single-spike pairing to induce tLTP (Fig. 1B, C). A possible mechanism by which depolarization could facilitate tLTP induction is by enhancing spike back-propagation in the dendrite (Sjöström et al., 2001; Stuart and Häusser, 2001). Indeed, it has been shown in neocortical layer 5 neurons that postsynaptic depolarization can convert tLTD into tLTP by boosting bAP (Sjöström and Häusser, 2006). This depolarization might be further enhanced by repetitive postsynaptic spiking if the membrane potential does not fully repolarize following each spike (Sjöström et al., 2001).

A minimum postsynaptic spike rate of 4–5 Hz was necessary for the single-spike pairing protocol to induce tLTP. A similar frequency dependence of spike pairing was demonstrated in the first report of spike timing-dependent potentiation in neocortical neurons (Markram et al., 1997). Consistent with the results in neocortical neurons, we found that single-spike pairing during postsynaptic membrane depolarization was ineffective with pairing frequencies of 1 or 3 Hz (Fig. 1D, E) but effective at 5 Hz (Fig. 1B, C). This suggests that tLTP induction in older hippocampus is pairing frequency-dependent. We found that it is the postsynaptic spike frequency that is important for tLTP induction since pairing 1 Hz presynaptic stimulation with single postsynaptic spikes at 5 Hz could induce tLTP (Fig. 2A) whereas pairing 5 Hz presynaptic stimulation with single postsynaptic spikes at 1 Hz failed to induce tLTP (Fig. 2B). This theta-frequency postsynaptic activity-requirement is interesting since CA1 pyramidal neuron membrane is known to resonate at theta-frequency range not only in the soma (Pike et al., 2000) but also in the apical dendrite with resonance strength increasing with distance from the soma (Narayanan and Johnston, 2008). Thus, it is possible that spike back-propagation at theta-frequency might have amplified the dendritic depolarization, allowing successful tLTP induction.

The third requirement we found to be important for tLTP induction at older CA3–CA1 synapses using single pre- and postsynaptic spikes is mGluR activation. Activation of mGluRs has been reported to reduce inhibition (Mannaioni et al., 2001), which could enhance bAP for tLTP induction. In addition to its direct effect on GABAergic interneurons, it is conceivable that mGluR activation could reduce GABA_A_ receptor-mediated inhibition via enhanced depolarization-induced suppression of inhibition (DSI) (Pitler and Alger, 1992) and endocannabinoid release (Maejima et al., 2001; Varma et al., 2001), which facilitates the induction of LTP (Carlson et al., 2002).

During tLTP induction we used a presynaptic stimulation frequency of either 5 Hz (Fig. 1B–C) or 1 Hz (Figs. 2A, 3–5). Previous work has established that low-frequency stimulation (LFS) of presynaptic input at 1–5 Hz alone induces LTD at CA3-CA1 synapses (Bear and Abraham, 1996; Dudek and Bear, 1992, 1993; Mulkey and Malenka, 1992). LFS activates mGluRs, and some forms of LFS-induced LTD require activation of mGluRs (Bolshakov and Siegelbaum, 1994; Fan et al., 2010; Oliet et al., 1997). Therefore, the presynaptic stimuli used in our study most likely activated mGluRs, and might have been expected to induce LTD. However, pairing LFS of afferent input with postsynaptic spikes at a frequency of 5 Hz or more caused the induction of tLTP instead (Fig. 3). What mechanisms could account for the conversion of LTD into NMDA receptor-dependent tLTP? The most likely possibility is that postsynaptic spikes increased the magnitude of the postsynaptic Ca^2+^ transient ([Ca^2+^]_i_), which has been suggested to determine the direction of synaptic plasticity, whereby low postsynaptic [Ca^2+^]_i_ induces LTD whereas a higher [Ca^2+^]_i_ induces LTP (Artola and Singer, 1993; Cavazzini et al., 2005; Zucker, 1999). A further boost of postsynaptic [Ca^2+^]_i_ could be provided by intracellular calcium stores, since presynaptic stimulation paired with 5–10 postsynaptic spikes at 30 Hz was shown to enhance IP_3_ receptor-mediated [Ca^2+^]_i_ increases elicited by group I mGluR activation (Nakamura et al., 1999). Therefore, activation of mGluRs by LFS paired with postsynaptic spikes at 5 or 10 Hz could have triggered mGluR-dependent Ca^2+^ release in the dendrite boosting the postsynaptic [Ca^2+^]_i_ beyond the threshold level required for LTP induction.

Group 1 mGluRs are comprised of mGluR1 and mGluR5 and strong expression of mGluR5 was observed in the dendrites of hippocampal CA1 pyramidal neurons (Lüscher and Huber, 2010; Lujan et al., 1996). In our experiments, application of the mGluR5 antagonist MPEP, or the more selective mGluR5 antagonist MTEP, blocked the induction of tLTP (Fig. 4C–D), which is, to our knowledge, the first direct indication of the involvement of mGluR5 in tLTP. Previously, MPEP has been reported to reduce high-frequency stimulation (HFS)-induced LTP both *in vitro* (Francesconi et al., 2004) and *in vivo* (Balschun and Wetzel, 2002) and mGluR5 knockout mice also displayed reduction in LTP as well as learning impairments (Jia et al., 1998; Lu et al., 1997). However, we cannot completely exclude the possibility that MPEP and MTEP might interfere directly with NMDA receptor function. Both DHPG and MPEP have been reported to act as antagonists on NMDA receptors (Contractor et al., 1998; Lea and Faden, 2006). However, we used 10 μM MPEP, and 40 μM MPEP was reported to have no effect on NMDA responses (Francesconi et al., 2004). Moreover, MTEP has an even greater selectivity at mGluR5 than that of MPEP (Lea and Faden, 2006), and would not be expected to have off-target effects at the concentration used here (500 nM). Consistent with this, we found no significant effect of MTEP on synaptically-evoked NMDA current (Fig. 4E). It therefore appears likely that the effects we observed of MPEP and MTEP are mediated through their antagonist effect on mGluR5.

The activation of group 1 mGluRs and their involvement in tLTP induction in our study is interesting since mGluR activation has been implicated in both LTP and LTD. It has been suggested that mGluR activation is required for HFS-LTP induction in CA1 pyramidal neurons (Bashir et al., 1993), that it can enhance HFS-LTP (McGuinness et al., 1991a, b), and that application of mGluR agonist alone can induce LTP (Bortolotto et al., 1995; Bortolotto and Collingridge, 1993, 1995). In addition, prior activation of mGluRs, called “priming” was shown to facilitate LTP (Cohen and Abraham, 1996; Cohen et al., 1998) and mGluRs have been suggested to serve as a molecular switch for LTP induction (Bortolotto et al., 1994, 2005). On the other hand, brief application of the mGluR agonist DHPG can induce LTD *in vitro* and *in vivo* (Fitzjohn et al., 1999, 2001; Huber et al., 2000; Huber et al., 2001; Kemp and Bashir, 1999; Lüscher and Huber, 2010; Manahan-Vaughan, 1997; Naie and Manahan-Vaughan, 2005; Palmer et al., 1997; Watabe et al., 2002). Consistent with these latter observations, we observed that bath application of DHPG depressed synaptic transmission in both test and control pathways (Fig. 5A), and the unpaired control pathway remained depressed. However, the paired pathway potentiated to a level similar to that following 1 Hz pre-10 Hz post tLTP induction paradigm (Figs. 3C and 4A). That is, simple pairing of presynaptic input with single postsynaptic spikes was sufficient to switch mGluR-mediated LTD to NMDA receptor-dependent tLTP (Fig. 5A, B), gated by mGluR5 (Fig. 5A, C). This result reconciles the two apparently conflicting results from field recordings in which application of mGluR agonist could result in both LTP and LTD. Application of DHPG might result in an increase of postsynaptic [Ca^2+^]_i_, which at low levels could induce LTD, whereas postsynaptic spikes might boost postsynaptic [Ca^2+^]_i_ sufficiently to convert LTD into LTP.

In conclusion, we show that tLTP can be induced by single spike-pairing at CA3–CA1 synapses in older hippocampus and that it requires postsynaptic depolarization as well as postsynaptic spikes at theta-frequency, which appears physiologically realistic. We suggest that this tLTP is gated by mGluR5, which is a first indication of its involvement in tLTP induction. Importantly, we show that physiological activation of mGluR by the induction paradigm, rather than pharmacological activation of mGluR, is sufficient for this gating function. These results suggest that the overall activity of the network influences the induction of tLTP, and during theta activity, cholinergic activation (Sugisaki et al., 2011) as well as mGluR activation could dynamically modulate tLTP induction.
